# Supplementary material for: Evaluating the RIST Molecular-Targeted Regimen in a Three-Dimensional Neuroblastoma Spheroid Cell Culture Model
Source: Cancers (Basel). 2023 Mar 14;15(6):1749. doi: 10.3390/cancers15061749 (PMC10046822; doi:10.3390/cancers15061749)

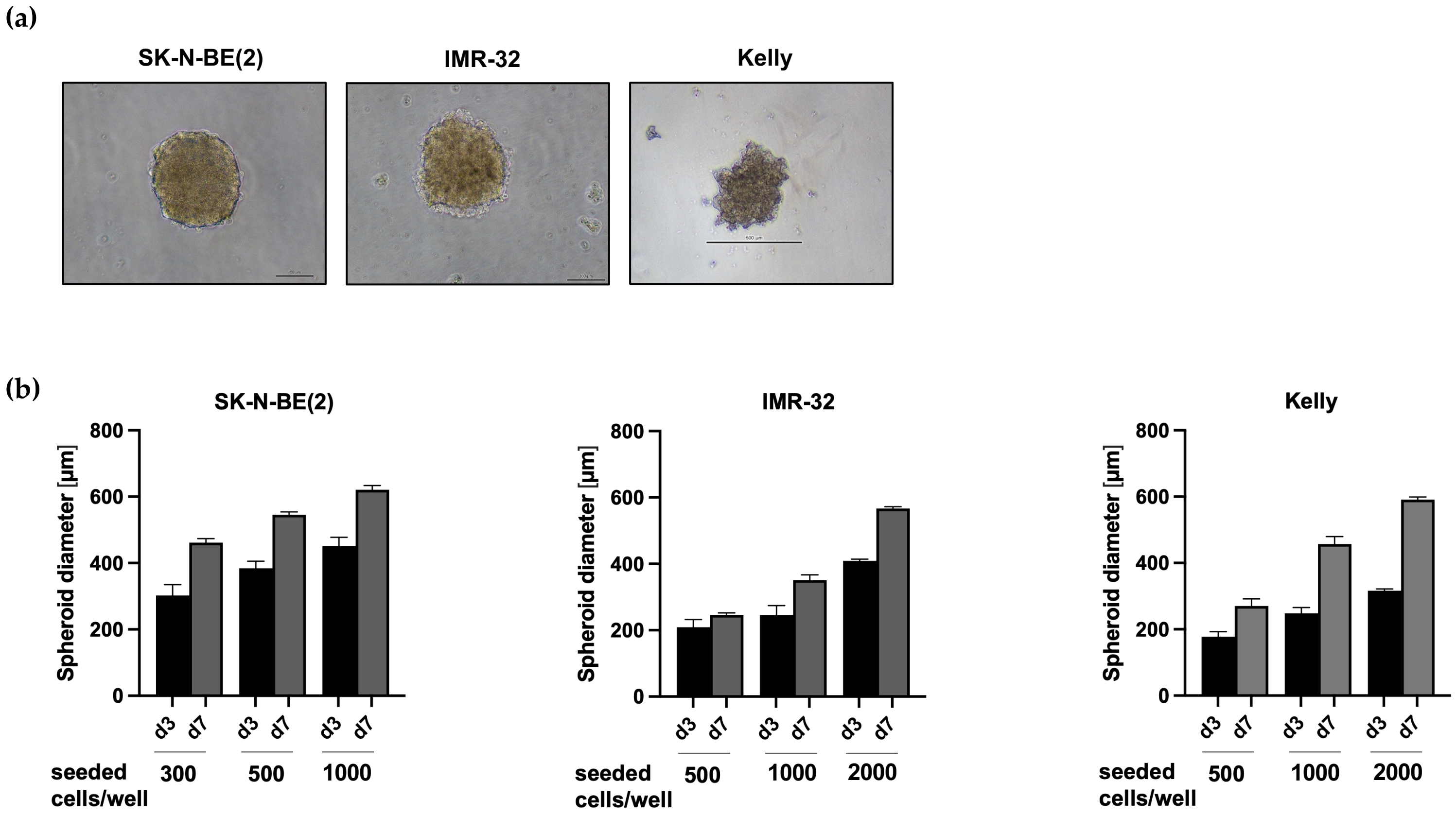

Figure S1: Establishment of spheroid culture conditions. ***(a)*** *Phase-contrast images of SK-N-BE(2) (500 cells/96-well seeded), IMR-32 (1000 cells/96-well seeded), and Kelly (2000 cells/96-well seeded) spheroids grown for 3 days on round bottom 96-well ULA plates. Scale = 100 µM, respectively 500µm for Kelly.* ***(b)*** *Analysis of the diameter of SK-N-BE(2) (left), IMR-32 (middle), and Kelly (right) spheroids seeded with increasing cell numbers on round bottom 96-well ULA plates for 3 days (d3) and 7 days (d7). At least two independent experiments (n=2) in triplicates were performed.*

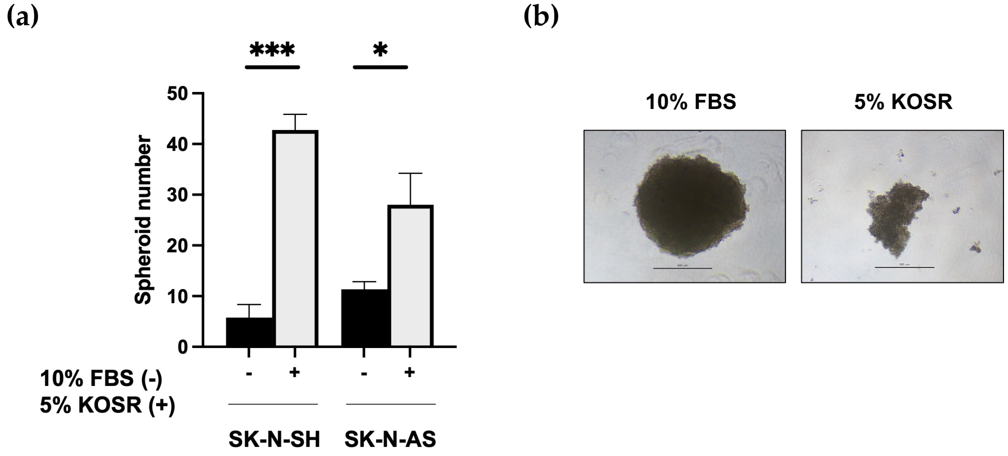

Figure S2: Establishment of spheroid culture conditions. (a) *Number of SK-N-SH and SK-N-AS (1000 cells/well seeded) spheroids grown with 10% fetal bovine serum (FBS) (–) or with 5% knock-out serum replacement (KOSR) (+) as growth supplement on a flat bottom 96-well ULA plate. Number of spheroids were counted on day 7. Three independent experiments (n=3) in triplicates were performed.* ***(b)*** *Phase-contrast images of Kelly spheroids comparing growth with 10% fetal bovine serum (FBS) (–) or 5% knock-out serum replacement (KOSR) (+) on round bottom 96-well ULA plates (4000 cells/well seeded) for 10 days, scale = 500 μM. Degrees of significance (unpaired student's t-test): * < 0.05 and *** < 0.001.*

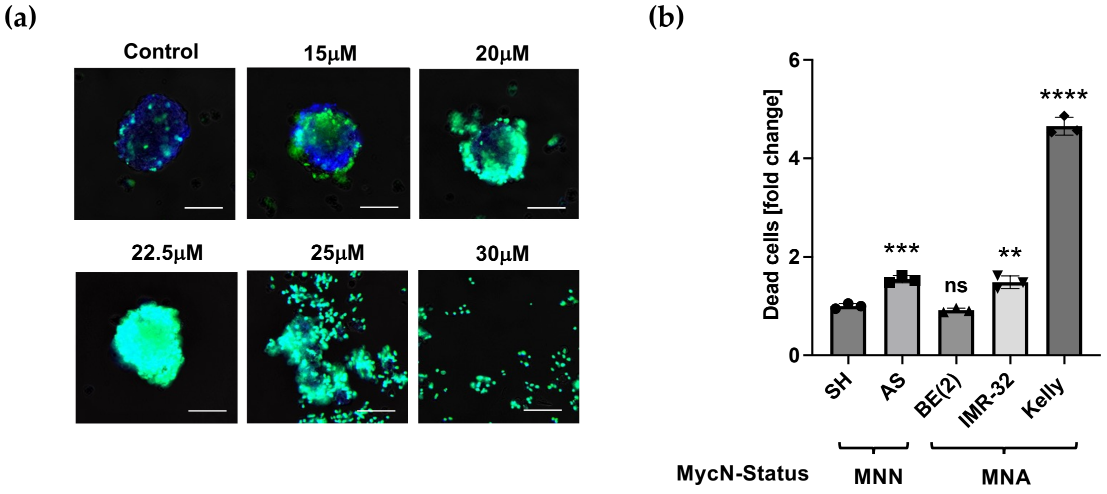

Figure S3: Spheroid characterization. ***(a)*** *Inducing cell death in spheroids (SH-N-SH) by treatment with high and increasing concentrations of Rapamycin for 72h followed by staining with NucBlue Live ReadyProbes Reagent (cell-permeable, fluorescent after DNA binding, ThermoFisher #R37605) and CellTox Green Dye (excluded from viable cells, but preferentially stains dead cell's DNA, Promega #G8741). Control: treatment with vehicle. Pictures were taken applying the ZOE Fluorescent Cell Imager (Bio-Rad), scale = 100 µM.* ***(b)*** *CellTox Green Dye staining and fluorescence measurement on day 7 after of SK-N-SH (SH), SK-N-AS (AS), SK-N-BE(2) (BE(2)), IMR-32, and Kelly spheroids were seeded as shown in Table 1 and grown on round bottom 96-well ULA plates. Fold change compared to CellTox Green Dye signal of SK-N-SH spheroids. MycN-non-amplified (MNN), MycN-amplified (MNA). Representative results from two independent experiments in triplicates. Degrees of significance (unpaired student's t-test): * < 0.05, ** < 0.01 and *** < 0.001.*

The following pages contain all original immunblot (IB) images as Supplementary Materials *Figure S4*

P-p70S6K (Thr389) and p70S6K, P-4E-BP (Ser65) and 4E-BP

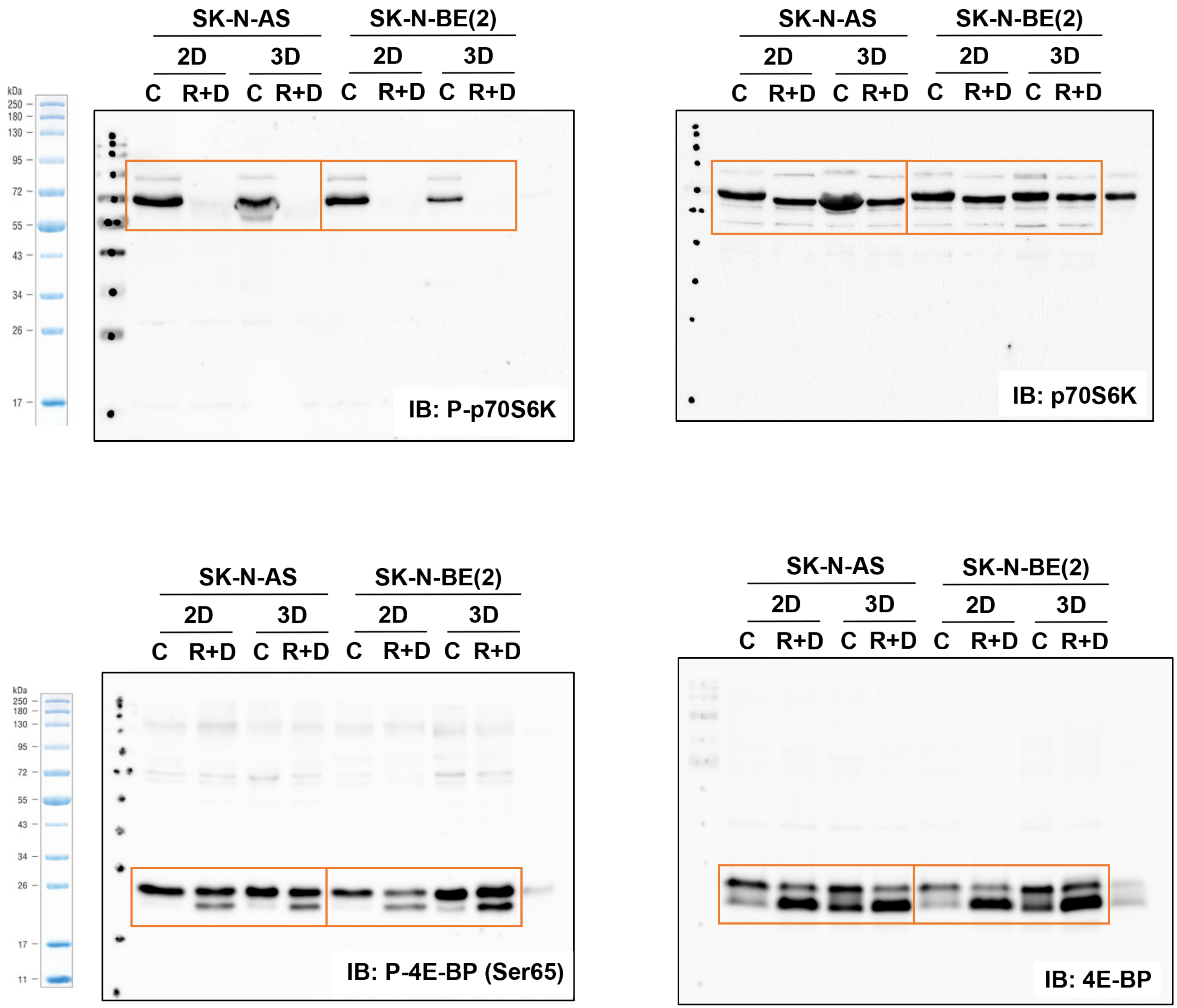

P-Src (Tyr416) and Src, GAPDH

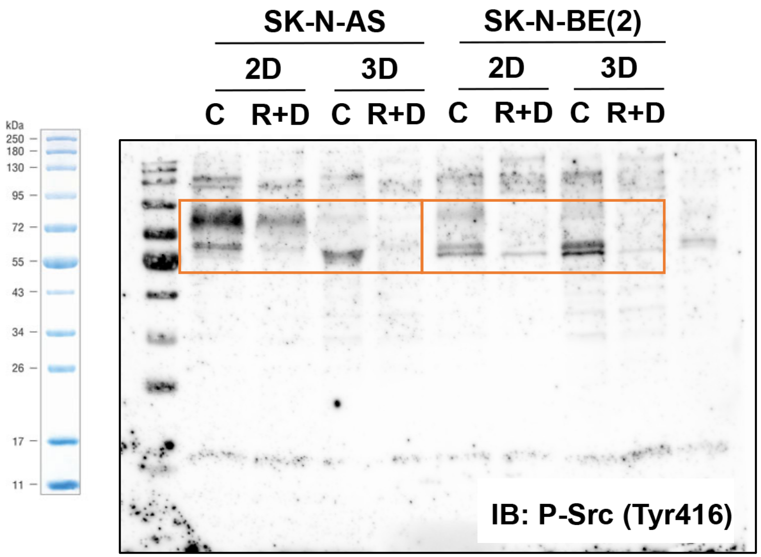

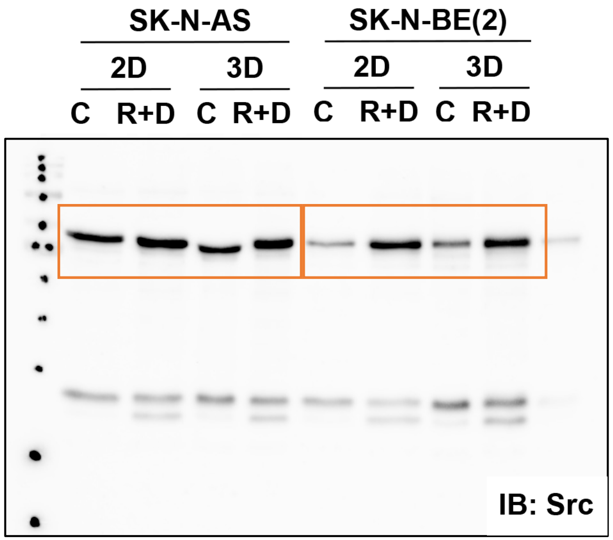

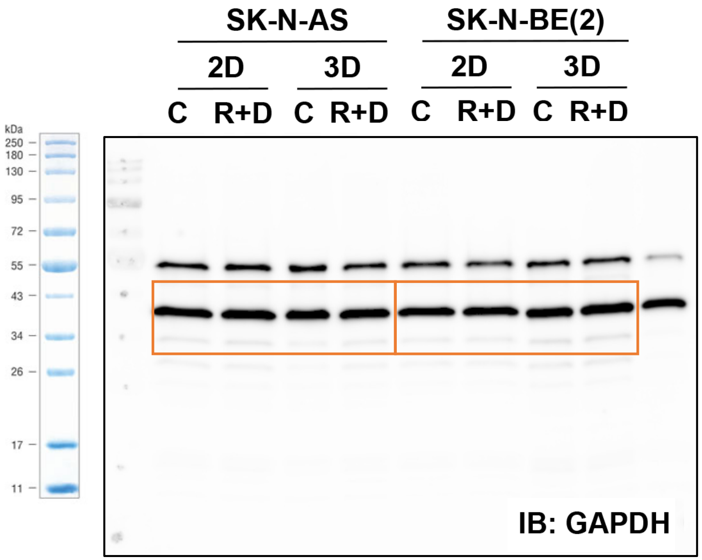

CXCR4, GAPDH

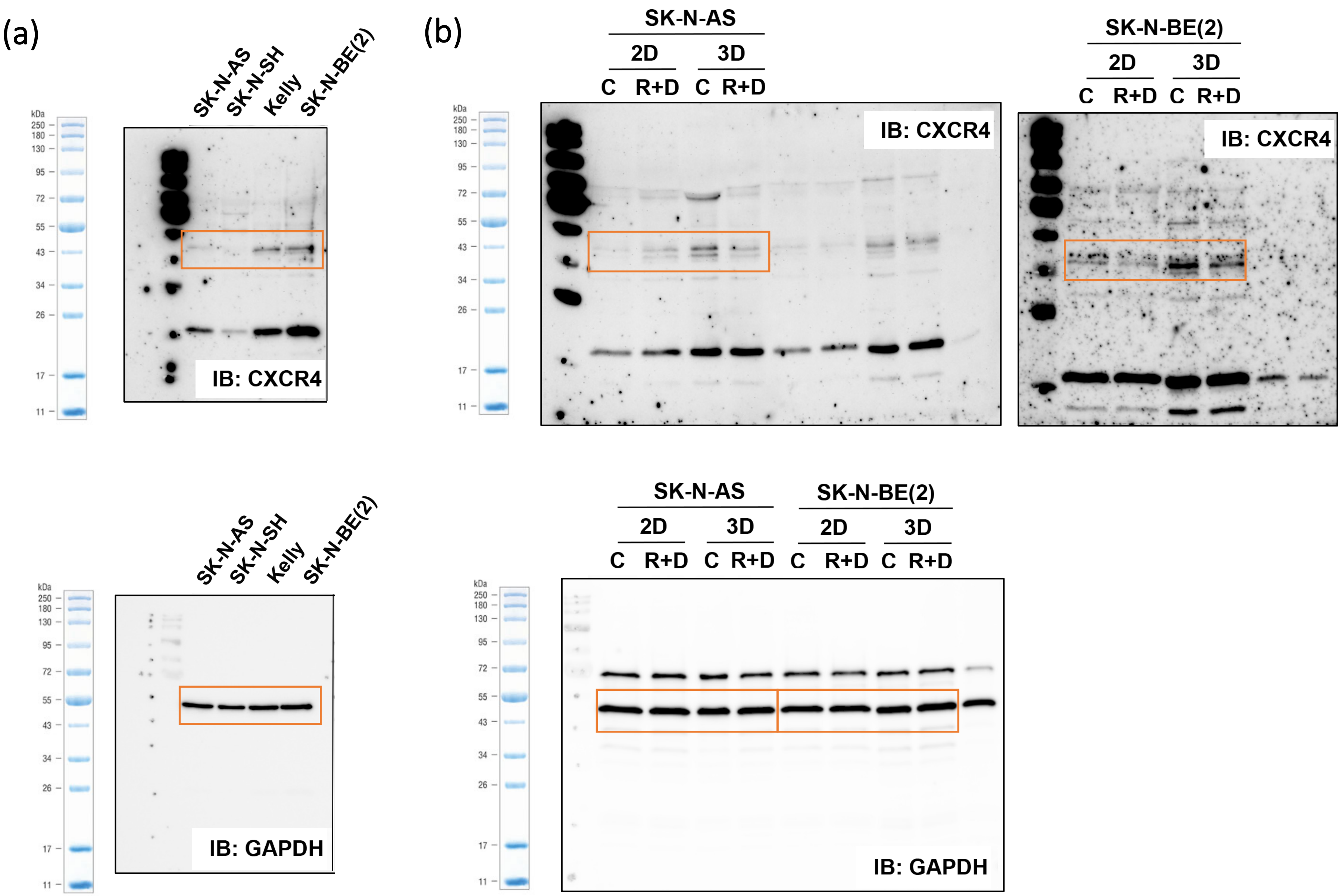
(a)
SK-N-AS
SK-N-SH
Kelly
SK-N-BE(2)
kDa
250
180
130
95
72
55
43
34
26
17
11
IB: CXCR4
IB: GAPDH
(b)
SK-N-AS
SK-N-BE(2)
2D
3D
C
R+D
IB: CXCR4
IB: GAPDH

BMI1 AND NANOG

(a)

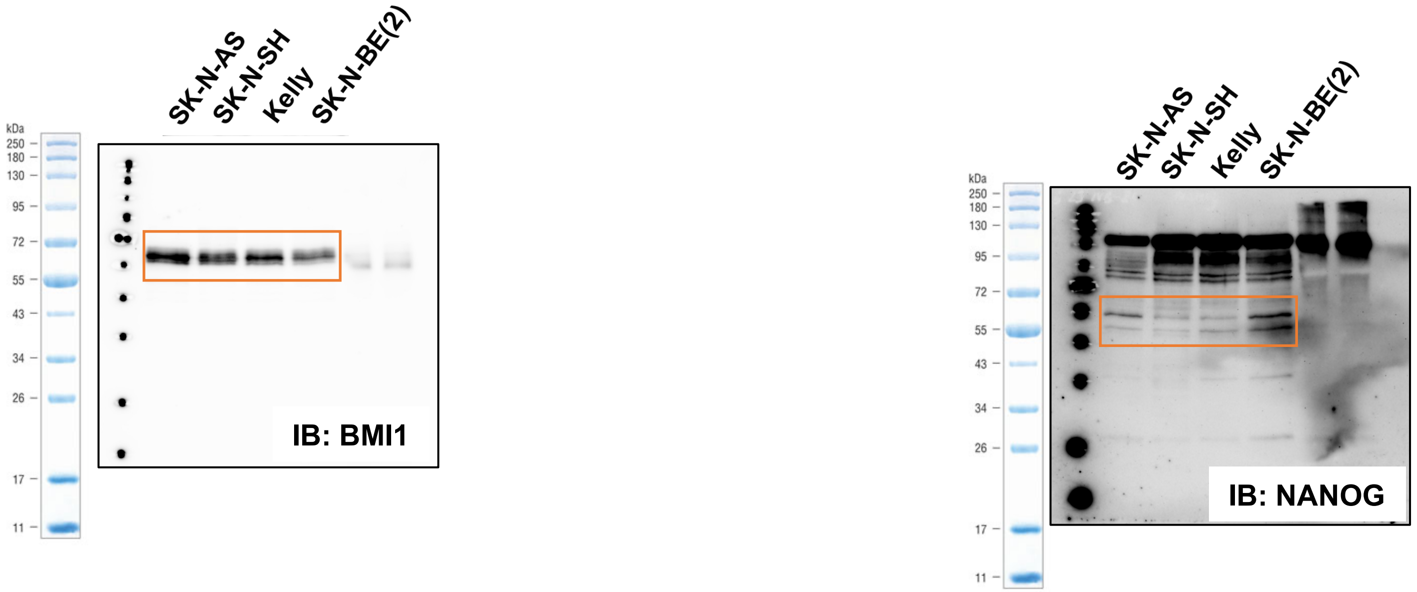

(b)

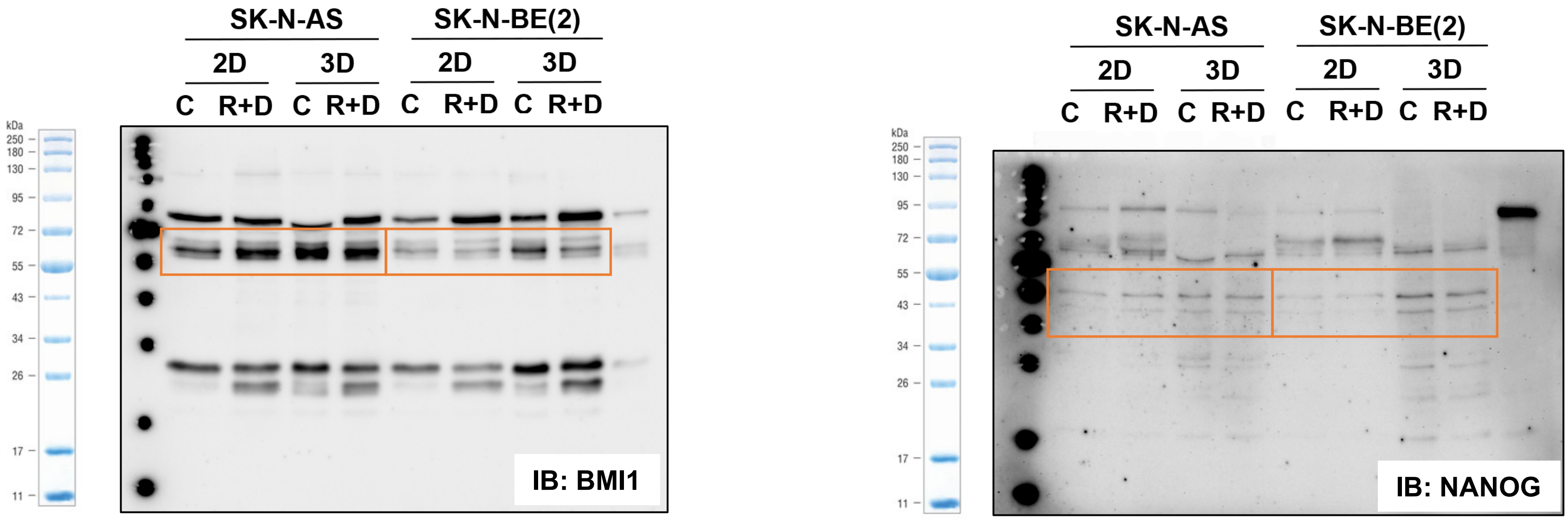

MycN, P-La (Thr 389) and La, GAPDH

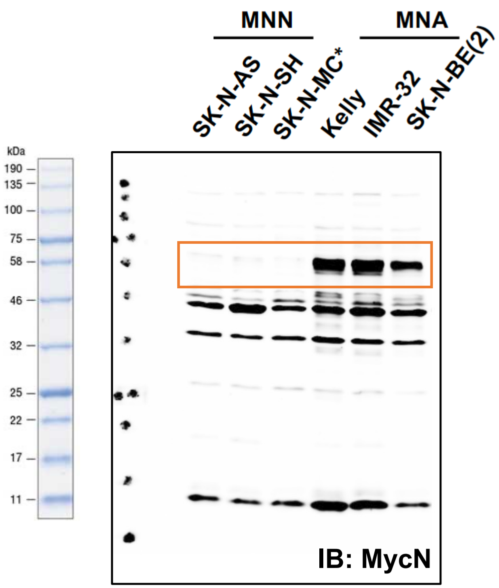

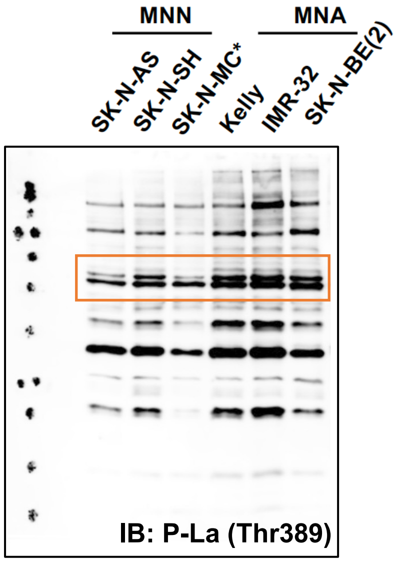

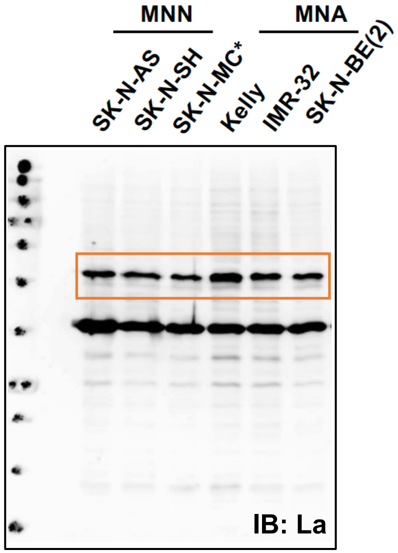

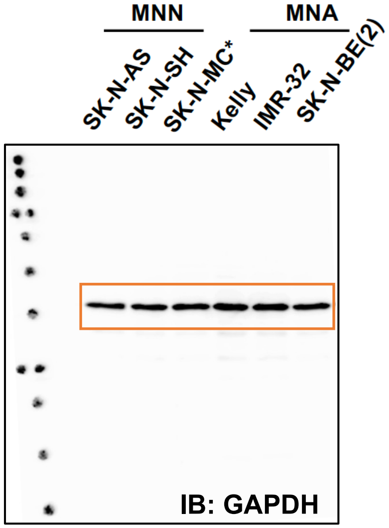

P-La (Thr 389) and La, GAPDH

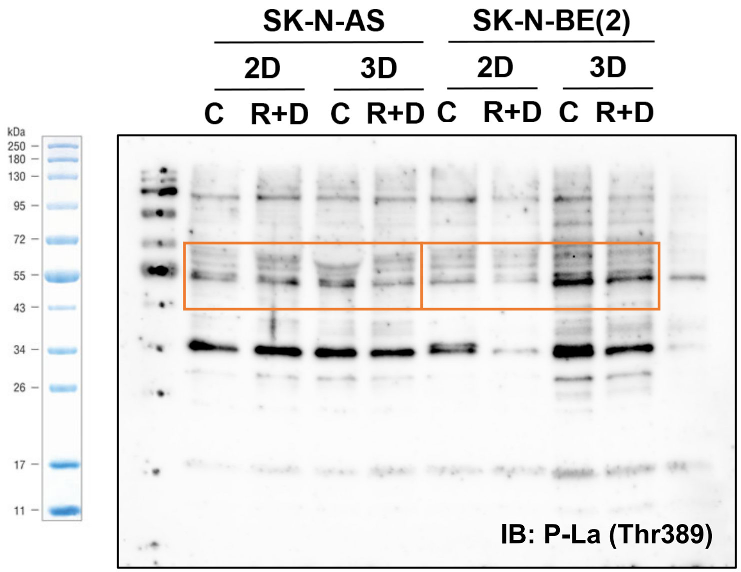

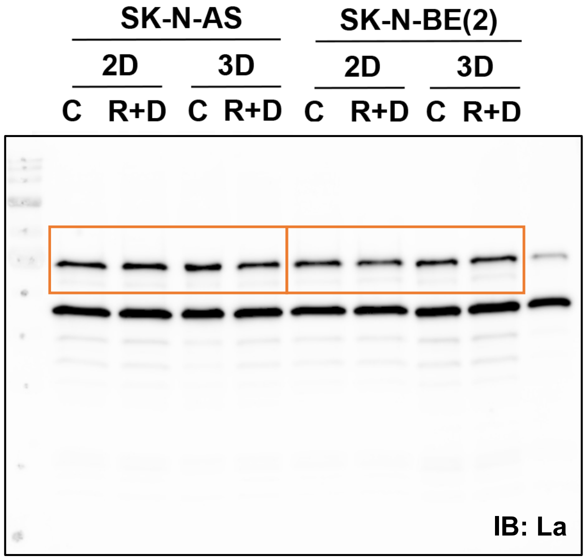

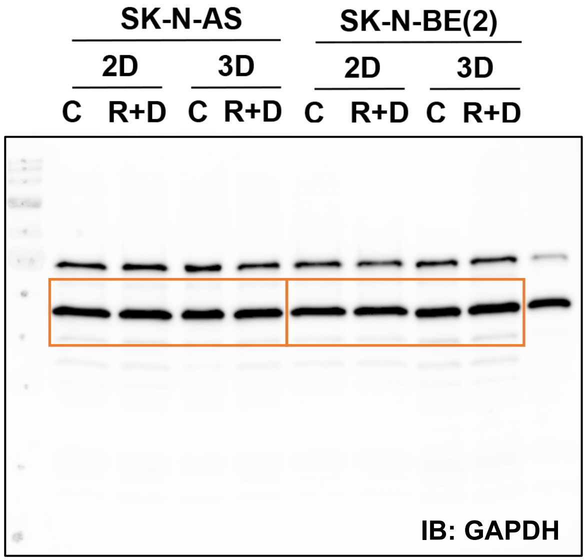

Supplement: Supplementary file 1 [file cancers-15-01749-s001.zip › cancers-2148969-supplementary.pdf]
